# Inhibitory effect of curcumin on testosterone induced benign prostatic hyperplasia rat model

**DOI:** 10.1186/s12906-015-0825-y

**Published:** 2015-10-22

**Authors:** Su Kang Kim, Hosik Seok, Hae Jeong Park, Hye Sook Jeon, Sang Wook Kang, Byung-Cheol Lee, Jooil Yi, Sang Yeol Song, Sang Hyub Lee, Young Ock Kim, Joo-Ho Chung

**Affiliations:** Kohwang Medical Research Institute, School of Medicine, Kyung Hee University, Seoul, 130-701 Republic of Korea; Department of Internal Medicine, College of Oriental Medicine, Kyung Hee University, Seoul, 130-702 Republic of Korea; Department of Urology, College of Medicine, Kyung Hee University, Seoul, 130-702 Republic of Korea; Development of Ginseng and Medical Plants Research Institute, Rural Administration, Eumseong, 369-873 Republic of Korea

**Keywords:** Curcumin, Benign prostatic hyperplasia, Testosterone, BPH

## Abstract

**Background:**

Benign prostatic hyperplasia (BPH) is one of the common male diseases, which is provoked by dihydrotestosterone (DHT) and androgen signals. Several studies showed that curcumin has various effects of prevention and treatment to diseases. We investigated whether curcumin may repress the development of BPH in male Wistar rats.

**Methods:**

Seven weeks male Wistar rats were and divided into 4 groups (normal group, BPH group, finasteride group, curcumin group; *n* = 8 for each group). In order to induce BPH in rats, rats were castrated and testosterone was injected subcutaneously everyday (s.c., 20 mg/kg). Rats in the curcumin group were treated 50 mg/kg, administered orally for 4 weeks. After 4 weeks, all rats were sacrificed and their prostate and serum were analyzed.

**Results:**

Compared to the finasteride group as positive group, the curcumin group showed similarly protective effect on BPH in histopathologic morphology, prostate volume. Results of immunohistochemistry and western-blot showed decreased expressions of VEGF, TGF-ß1, and IGF1 were also decreased in the curcumin group.

**Conclusions:**

These results suggested that curcumin inhibited the development of BPH and might a useful herbal treatment or functional food for BPH.

## Background

Benign prostatic hyperplasia (BPH) is a common male disease causing lower urinary tract distress in aging men [1]. Several studies suggested that BPH is a multifactorial disease [2], however, it has known that hormonal factor contributes to the hyperplastic growth of prostate [1].

Underlying etiology of BPH has not been completely identified, however, it has been well established that the prostate gland in the aging men is affected by androgens. Many researchers reported steroid 5-alpha reductase, which converts testosterone in serum into dihydrotestosterone (DHT) in target tissue, as most important factor in current BPH treatment [3]. DHT synthesized by steroid 5-alpha reductase increases as aging, hence, prostatic gland of aged man may be affected more by steroid 5-alpha reductase [4]. Conventional steroid 5-alpha reductase inhibitors, such as finasteride and dutasteride, were successful in the treatment of hyperplastic growth of prostate, however, these drugs were responsible for adverse effects, such as gynecomastia, dizziness, upper respiratory infections, headache, and chest pain [5, 6]. Such effect may limit the use of conventional drugs for BPH, however, might be avoided by commonly used safe agents.

Curcumin is widespread, inexpensive, and highly safe to human [7]. It is polyphenolic natural compound from Curcuma longa [8], with diverse drug activities against aging related events, including dermatologic changes [9], retinal diseases [10], Parkinson’s disease (PD) [11], renal antioxidative effect [12], ischemic oxidative damages in diverse organs [13–15], and cancers [16]. Curcumin analogues may facilitate degradation of androgen receptor (AR) in prostate cancer [17], and may induce apoptosis of prostate cancer cell by IkBalpha, c-Jun and androgen receptor [18]. Curcumin shows suppressive effect on HIF1a [19, 20], which was suggested as a key molecule for transition from prostatitis to BPH [21]. Moreover, it was proven to be a potent TNF blocker [7], and its antioxidant effect may suppress LOX1 [22]. Administration of curcumin was investigated as liposomal-formulated curcumin in Parkinson’s disease (PD) [11], and lecithinized curcumin delivery system in BPH [23].

These previous reports indicate that curcumin may have protective role in BPH, however, effect of curcumin on BPH has not been investigated, which is responsible for the synthesis of DHT, an active metabolite of testosterone. Therefore, we investigated whether the curcumin has protective on testosterone induced BPH rat model.

## Methods

### Preparation of the curcumin

The chemical structure of curcumin was purchased from SAMCHUN (#121703 Pyongtack, Korea).

### Animals

The animals used in this study were 7 weeks male Wistar (Central Lab Animal Inc, Korea) with an average body weight of 250 ± 10 g. The animal room was maintained at 22 ± 2 °C and at 40 ~ 70 % relative humidity. Room lighting consisted of 12 h periods of light and dark. All experiments were carried out according to the protocols approved by the Animal Care Committee of the Animal Center at Kyung Hee University and in accordance with guidelines from the Korean National Health Institute of Health Animal Facility.

### Induction of BPH and treatments

After orchiectomy, the rats were divided into four groups (*n* = 8). BPH was induced by subcutaneous injection of testosterone (20 mg/kg) for 4 weeks: (A) a normal group; (B) BPH that induced testosterone group, injected subcutaneously; (C) curcumin group that treated 50 mg/kg, administered orally for 4 weeks; (D) finasteride group that treated 1 mg/kg, administered orally, as a positive anti-BPH drug and was purchased from Sigma-Aldrich (St Louis, MO, USA). The dosage of curcumin (50 mg/kg) referred to previous study [7]. All materials were administered to animals once daily for 4 weeks, and body weight was measured weekly. After 4 weeks, all animals were fasted overnight. Blood was collected in EDTA tubes, placed on ice, serum immediately was separated and stored at −20 °C. After animals were sacrificed, fresh prostate was stored in formaldehyde solution for light microscopic observation. The rest of the prostate were stored at −70 °C for the later analysis.

### Blood collection and biochemical analysis

At the end of the experiment, the food was removed and experiments were performed between 9 AM and 12 PM. Blood samples were obtained in serum separating tube (SST) from the heart of rat at the end of the experiment. Blood samples were centrifuged at 3000 × g for 15 min, at 4 °C and serum was obtained and stored at −70 °C before analyzed biochemical test. Glucose, total protein, GOT (glutamic oxaloacetic transaminase), and GPT (glutamic pyruvic transaminase), were analyzed by Greenlab (Seoul, Korea).

### Histopathological examination

Fixed prostate tissue embedded in paraffin wax was cut into 5 μm thick sections. Then the tissues were stained by Harris’ hematoxylin-eosin according to standard procedure. The sections were mounted and cover slipped using mounting solution. Size and thickness of epithelial cell in ventral lobe of prostate tissue was assessed in order to identify effect of curcumin on BPH.

### Immunohistochemistry

Immunostaining was performed on 4 μm sections after deparaffinization. Antigen retrieval was performed in citrate buffer pH 6.0 with 95 °C for 10 min prior to peroxides quenching with 3 % H_2_O_2_ in PBS for 10 min. Then sections were washed with PBS and preblocked with normal goat or rabbit serum for 10 min. In the step of primary antibody reaction, slides were incubated, respectively, with anti-VEGF (Santa Cruz, CA, USA) in a 1:200 dilution, anti-TGF-β1 (Santa Cruz, CA, USA in a 1: 200 dilution, and anti-IGF-1 (Santa Cruz, CA, USA) in a 1: 200 dilution for overnight at 4 °C. Then, the sections were incubated with biotinylated secondary antibodies (1: 1000) for 1 h. Following a washing step with PBS, the streptodavidin-HRP was applied. Finally, the sections were rinsed in PBS, developed with diaminobenzidine tetrahydrochloride substrate (DAB) in 10 min. At least three random region of each section were examined at × 100 and × 400.

### Western-blot

Prostate tissues were homogenized by a tissue homogenizer in protein lysis buffer. After centrifuge at 12,000 rpm, protein was extracted and protein concentration was determined using Bradford protein assay. Proteins were transferred membranes and membranes were incubated with the antibodies used for western-blot analysis were anti-VEGF (Santa Cruz, CA, USA) in a 1:200 dilution, anti-TGF-β1 (Santa Cruz, CA, USA in a 1 : 200 dilution, and anti-IGF-1 (Santa Cruz, CA, USA) for overnight. Then, HRP-conjugated secondary antibody (1:5000, Pierce Chemical) for 1 h at room temperature (RT) and membrane was developed with ECL western blotting detection reagents (GE Healthcare Biosciences, Pittsburgh, PA, USA).

### Statistical analyses

All of the values showed as the mean ± S.E. Significant difference among the groups was statistically performed using a one-way analysis of variances (ANOVA), followed by a non-parametric post Tukey test. All *p* values are two-tailed, and significance was set at *p* < 0.05. All statistical analysis was performed using the SPSS for windows, version 21.0 (SPSS Inc.).

## Results

### Measurement of weight change

Body weight change shows in Table 1. Final body weight was measured after 4 weeks. The final body weight in normal, BPH, curcumin, and finasteride groups was increased compared with initial body weight (normal group, 69. 06 %, BPH group, 68.49 %, curcumin group, 67.93 %, and finasteride group 68.12 %, respectively). The differences among each groups did not show significant difference (*p* > 0.05).

**Table 1:** Effects of *curcumin* on body weight in each group

	Normal	BPH	Curcumin	Finasteride
Body weight (g)				
Initial weight	250	250	250	250
Final weight	362.23 ± 5.72	365.50 ± 6.26	368.34 ± 5.43	367.50 ± 6.75
Total body weight gains for 30 days	112.23 ± 5.72	115.50 ± 6.26	118.34 ± 5.43	117.50 ± 6.75

Data are presented as mean ± S.E. (*n* = 8)

Normal, normal group; BPH, testosterone induced BPH group; c*urcumin*, testosterone induced BPH with *curcumin* (200 mg/ml) group; finasteride, testosterone induced BPH with finasteride (1 mg/ml) group

### Glucose, total protein, AST, and ALT level in serum level

The BPH group demonstrated decrease glucose level in serum than the normal group (Table 2). Curcumin treatment group showed effect on plasma glucose. AST level in serum did not significantly different among groups. However, ALT level in curcumin group showed higher than other groups. And, total protein level in serum in the BPH group was slightly increased, compared with the normal group. However, differences among the groups were not statically significant (*p* > 0.05). The total protein of curcumin group showed decrease compared with that of the normal group.

**Table 2:** The effect of *curcumin* in each group

	Normal	BPH	Curcumin	Finasteride
Glucose (mg/dl)	137.00 ± 8.09	115.89 ± 3.48^#^	153.60 ± 3.18**	128.58 ± 5.79
Total protein (g/dl)	5.72 ± 0.06	5.92 ± 0.06	5.65 ± 0.12	5.85 ± 0.09
AST (GOT) (U/L)	88.86 ± 6.50	97.67 ± 9.69	101.20 ± 6.86	101.43 ± 4.92
ALT (GPT) (U/L)	28.43 ± 1.80	28.12 ± 1.53	36.00 ± 2.22^#,^ *	28.86 ± 0.80

Data are presented as mean ± S.E. (*n* = 8)

^#^ *P* < 0.05 compared with normal group; *P < 0.05, **P < 0.01, compared with BPH group

### Prostate ratio according to volume and weight of the prostate

Prostate weight, volume, and weight ratio show in Table 3. Prostate weight of the BPH group (1.36 ± 0.03 g, mean ± S.E.) was significantly increased compared with that of the normal group (1.04 ± 0.04 g), and the administration groups of curcumin and finasteride treatment group were significantly decreased than that of the BPH group, 0.91 ± 0.05 g and 0.73 ± 0.03 g, respectively. The prostate volume of BPH group (1.73 ± 0.19 cm^3^) was significantly higher than that of normal group (1.13 ± 0.12 cm^3^), and the administration groups of curcumin and finasteride group were significantly lower than that of BPH group, 0.67 ± 0.06 cm^3^ and 0.86 ± 0.08 cm^3^, respectively. Finally, prostate weight ratio of the BPH group (0.40 ± 0.03 mg/100 g of BW) was significantly higher than that of the normal group 0.28 ± 0.02 mg/100 g of BW), and the administration groups of curcumin group and finasteride group were significantly lower than that of the BPH group, 0.22 ± 0.02 mg/100 g of BW and 0.27 ± 0.02 mg/100 g of BW, respectively.

**Table 3:** Prostate weight, prostate volume, and prostate weight ratio in each group

	Normal	BPH	Curcumin	Finasteride
Prostate weight (g)	1.04 ± 0.04	1.36 ± 0.03 ^###^	0.91 ± 0.05 ***	0.73 ± 0.03 ***
Prostate volume (cm^3^)	1.13 ± 0.12	1.73 ± 0.19 ^###^	0.67 ± 0.06 ***	0.86 ± 0.08 ***
Prostate weight ratio (mg/100 g of BW)	0.28 ± 0.02	0.40 ± 0.03 ^###^	0.22 ± 0.02 ***	0.27 ± 0.02 ***

Data are presented as mean ± S.E. (*n* = 8)

^###^ *P* < 0.001 compared with the normal group; ***P < 0.001 compared with the BPH group

### Histological morphology of the prostate

**Fig. 1:**
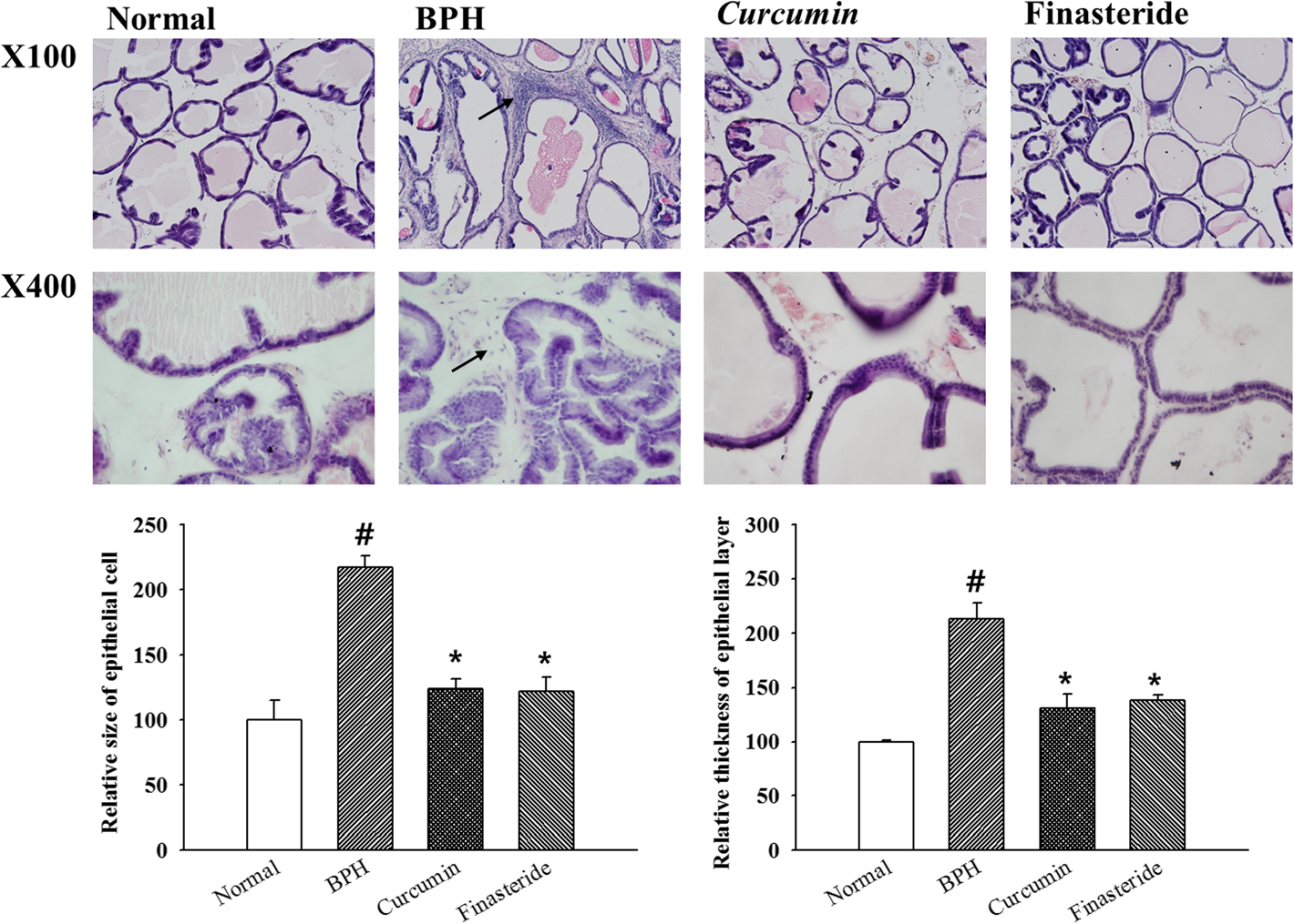
H&E stains of prostate tissue in each group (× 100 and × 400). Arrowheads indicate interacina fibrosis in ventral lobe of prostate tissue. # *P* < 0.05 compared with the normal group; * *P* < 0.05 compared with the BPH group

In order to examine the effects of the administration of curcumin on histological morphology of prostate tissue, H&E staining was performed. Prostate of rats generally consists of three distinct including dorsal lobe, lateral lobe, and ventral lobe. We analyzed ventral lobe of prostate tissue. As shown in Fig. 1, the histological morphology of the prostate tissue in the BPH group was abnormal. The connective tissue in prostate was increased oval shape and size. The epithelial cell layer and lumen space in the BPH group were increased than those of the normal group. And there was interacinar fibrosis in ventral lobe of rats in the BPH group. The decreases in hyperplasia and the epithelial layer thickness were observed in the curcumin group and the finasteride group as positive group compared with those of the BPH group (*p* < 0.05).

### Expression of VEGF, TGF-ß1, and IGF1 of prostate tissue

**Fig. 2:**
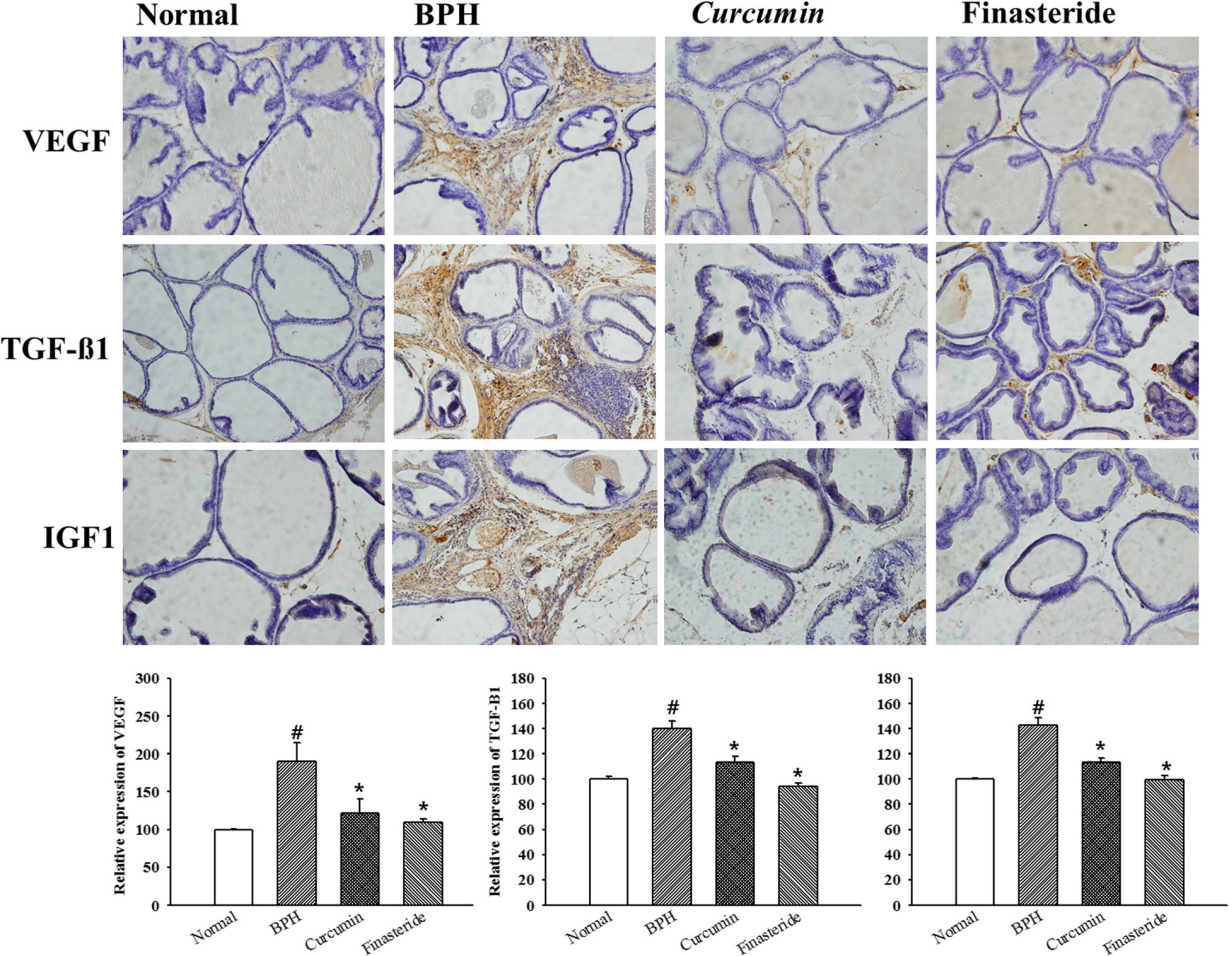
The expressions of vascular endothelial growth factor A(VEGF), transforming growth factor, beta 1(TGF-ß1), and insulin-like growth factor 1(IGF1) by immunohistochemistry in each group (× 100). # *P* < 0.05 compared with the normal group; * *P* < 0.05 compared with the BPH group

**Fig. 3:**
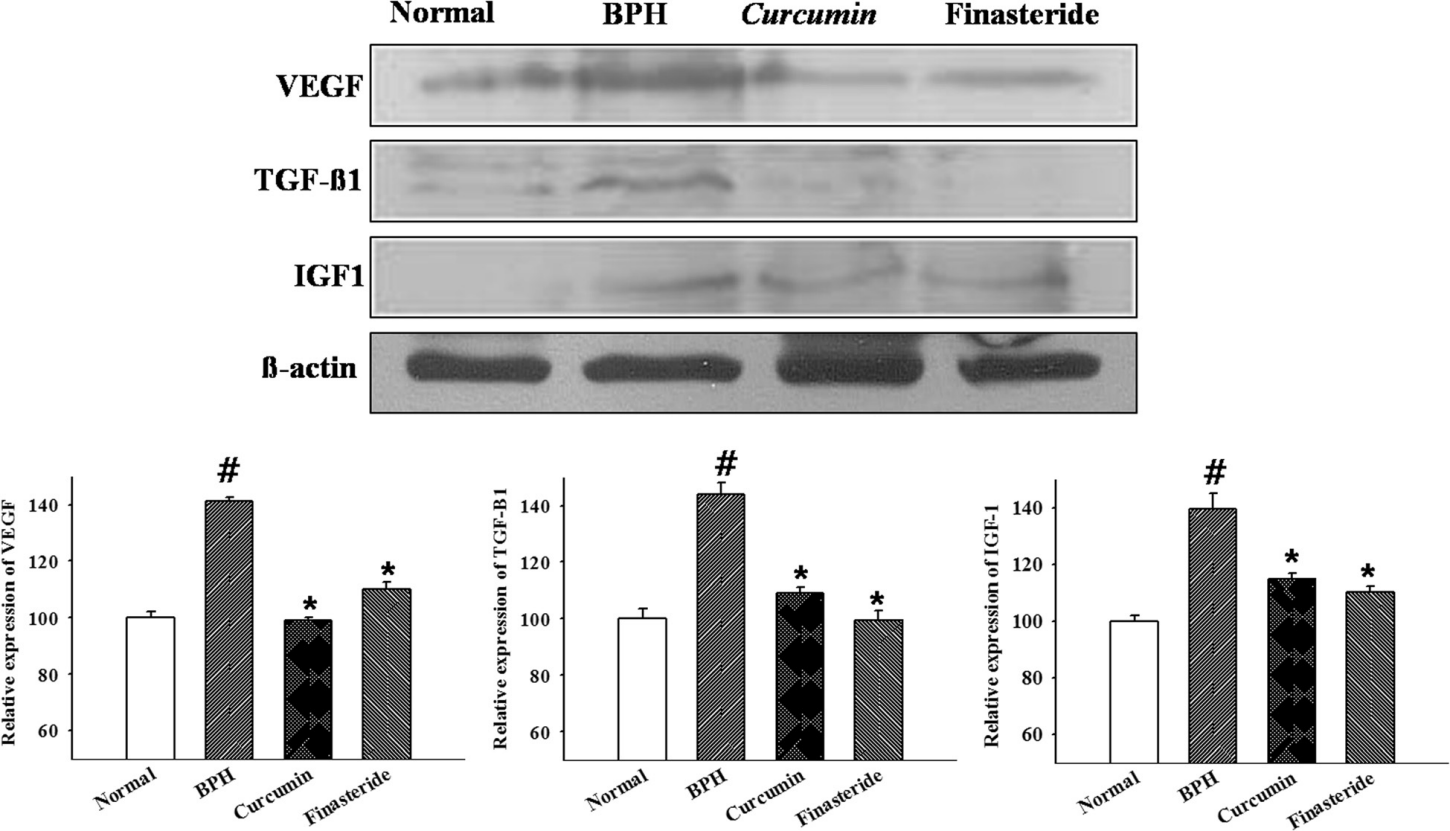
The expressions of vascular endothelial growth factor A(VEGF), transforming growth factor, beta 1(TGF-ß1), and insulin-like growth factor 1(IGF1) by western-blot in each group. # *P* < 0.05 compared with the normal group; * *P* < 0.05 compared with the BPH group

In order to examine the effects of the administration of curcumin on expression of growth factors in prostate tissue, IHC and western-blot were performed. As shown in Figs 2 and 3, the protein expressions of growth factors (VEGF, TGF-ß1, and IGF1) in the BPH group were increased than those of the normal group (*p* < 0.05). These proteins expressions of VEGF, TGF-ß1, and IGF1 both curcumin group and finasteride group showed decrease compared with those of the BPH group (*p* < 0.05).

## Discussion

In previous researches, blockade of steroid 5-alpha reductase was associated with inhibition of development of BPH [1]. Previous evidences for inhibition effect on BPH development in rats were found by reduction of prostate size or decreased histopahthologic morphology of BPH [21, 24].

Prostate require high levels of steroid androgens even for the maintenance of tissue architecture and AR [25]. Testosterone and DHT may influence cell signals by AR, which is nuclear sex steroid hormone receptor, and plays a role in activation of growth factors [4, 26]. Such role in the prostate has permissive effect in transformation from normal prostate to BPH or prostatic cancer [27–29]. Whether which of AR-activated growth factors may affect BPH development may be ambiguous, however, there were some previous evidences that decreased expressions of VEGF, TGF-ß1 [30, 31], and IGF1 [32, 33] were associated with inhibition of hyperplastic growth of prostate [29]. VEGF may be associated with angiogenic growth in hyperplasia [34]. Stromal extracellular matrix (ECM) accumulation is implicated in hyperplastic growth of BPH [26, 35, 36], and TGF-ß1 may affect the process of ECM deposition and inflammatory signals [37]. And last, IGF signaling was indicated to be responsible for the prostatic enlargement [38] and LUTS with obesity and diabetes [33].

It has been also known that such factors may be inhibited by curcumin. Curcumin may inhibit mitogenic effect of IGF1 and IGF1R signals in hypertrophic cells [39, 40]. Moreover, it may regulate various molecular targets including EGF, PDGF, VEGF, NF-kB, and STAT3 in cancers [41]. Regulation of VEGF by curcumin is seen in age-related macular degeneration model of mouse [42]. TGF-beta responses induced by cellular damages [43] and T cell-dependent inflammatory stress [44] may be reduced by curcumin. And chronic immune-mediated stress may be ameliorated by curcumin [45, 46]. These previous reports suggest that curcumin may have some beneficial effects on aging-related changes of various body organs, including prostate [18].

In the present study, curcumin showed positive effect of morphology in prostate ratio and histopathology in BPH rat model. Moreover, curcumin showed decreased expression of VEGF, TGF-ß1, and IGF1 when compared to testosterone induced BPH group. Our result is in concordance with previous reports that curcumin inhibited the expression of the growth factors, and that curcumin may inhibit androgen effect on prostate.

## Conclusion

In summary, curcumin significantly lower prostate weight and prostate volume. And curcumin treatment decreased expressions VEGF, TGF-ß1, and IGF1 among growth factors in prostate tissue.

These findings suggested that curcumin inhibited the development of BPH and might by a useful herbal treatment.

## Abbreviations

BPH: Benign prostatic hyperplasia
DHT: Dihydrotestosterone
AR: Androgen receptor
PD: Parkinson’s disease
GOT: Glutamic oxaloacetic transaminase
GPT: Glutamic pyruvic transaminase
ANOVA: One-way analysis of variances
IHC: Immunohistochemistry
